# The Incidental Finding of a Persistent Left Superior Vena Cava: Implications for Primary Care Providers—Case and Review

**DOI:** 10.1155/2015/198754

**Published:** 2015-01-06

**Authors:** Loren Garrison Morgan, Jonathan Gardner, Joe Calkins

**Affiliations:** Section of Cardiology, Department of Internal Medicine, The Medical College of Georgia at Georgia Regents University and Charlie Norwood VA Medical Center, 1120 15th Street, BBR 6518, Augusta, GA 30912, USA

## Abstract

Persistent left superior vena cava (PLSVC) is the most common thoracic venous anomaly and is a persistent congenital remnant of the vena caval system from early cardiac development. Patients with congenital anomalous venous return are at increased risk of developing various cardiac arrhythmias, due to derangement of embryologic conductive tissue during the early development of the heart. Previously this discovery was commonly made during the placement of pacemakers or defibrillators for the treatment of the arrhythmias, when the operator encountered difficulty with proper lead deployment. However, in today's world of various easily obtainable imaging modalities, PLSVC is being discovered more and more by primary care providers during routine testing or screening for other ailments. Given the known association between anomalous venous return and the propensity for cardiac arrhythmias, we review the embryology of PLSVC and the mechanisms by which it leads to conduction abnormalities. We also provide the practitioner with recommendations for certain baseline cardiac observations and suggestions for proper surveillance in hopes that better understanding will reduce unnecessary and potentially harmful testing, premature subspecialty referral, and unneeded patient anxiety.

## 1. Introduction

**Figure 1:**
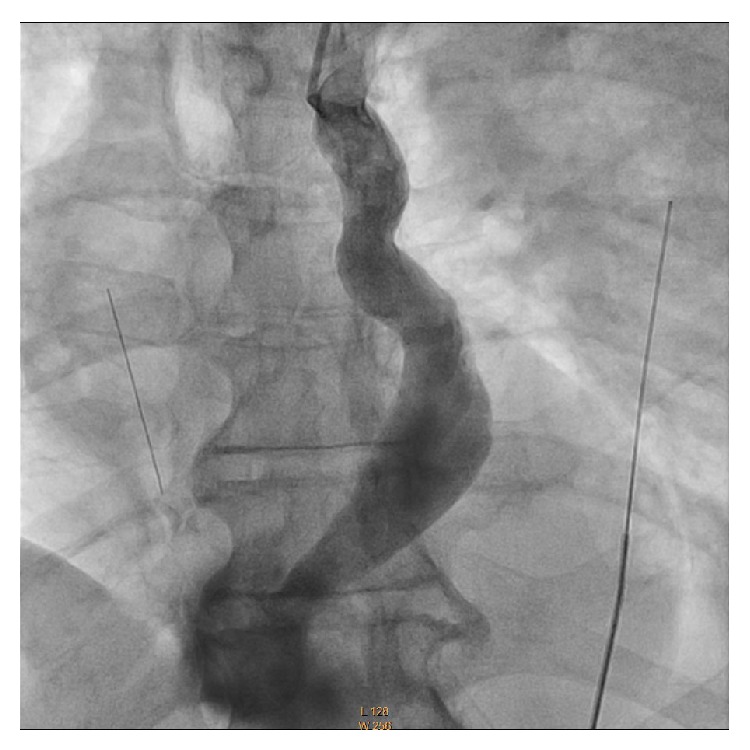
Venous angiogram showing contrast coursing through the persistent left superior vena cava and merging with the coronary sinus before emptying into the right atrium.

**Figure 2:**
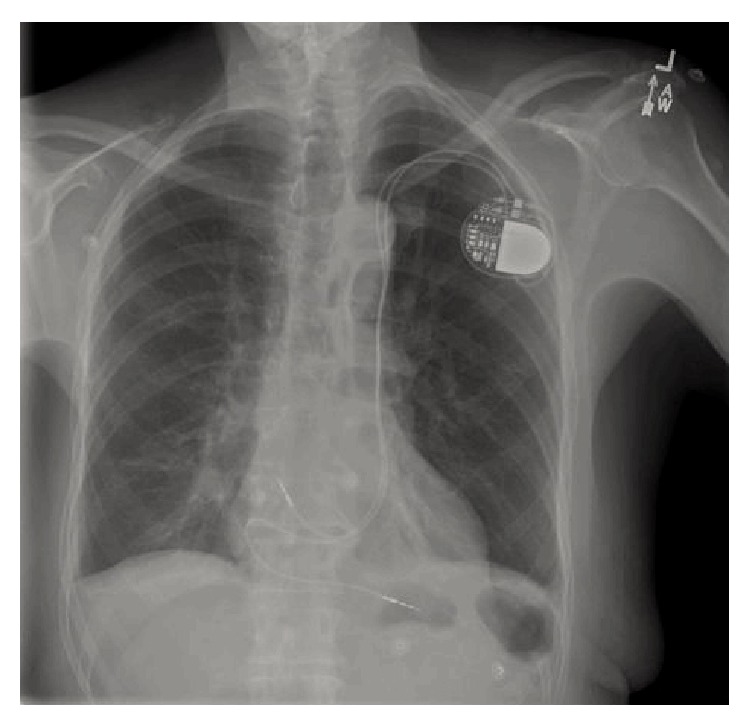
Chest X-ray showing postprocedure implantation of a dual chamber pacemaker, with leads traversing through a persistent left superior vena cava.

**Figure 3:**
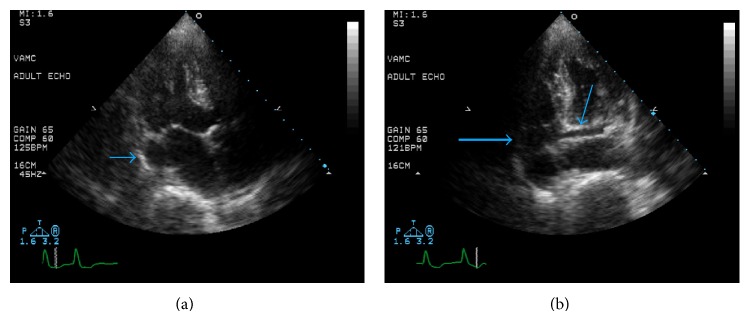
(a) Echocardiogram showing an off-axis longitudinal view of a portion of the persistent left superior vena cava (arrow) traversing under the left atrium on its course to the coronary sinus. (b) Echocardiogram showing an off-axis cross-sectional view of persistent left superior vena cava (large arrow) before it terminates in the dilated coronary sinus (small arrow).

An 84-year-old man presented for evaluation of dizziness and difficulty with ambulation and was found to have a pulse rate in the 30s. Electrocardiogram (ECG) showed sinus rhythm, complete heart block, and an escape rhythm with left bundle branch block morphology. Cardiac catheterization revealed nonobstructive coronary disease. In the CCU, the patient had various arrhythmias including atrial fibrillation with rapid ventricular rate and sinus rhythm with varying degrees of AV block. Due to continued AV block, placement of a permanent pacemaker (PM) was arranged. Preprocedure echocardiogram demonstrated normal left ventricular (LV) size and systolic function left atrial enlargement and a dilated coronary sinus, which at the time were thought to be an incidental finding. However, during implantation of his PM, there was difficulty with passage of the leads from his left subclavian access site. The leads were withdrawn and angiography was performed, showing a persistent left superior vena cava (PLSVC) which drained the left subclavian vein, and joined with the coronary sinus to empty into the right atrium (RA) (Figure 1). Once the anatomy was realized, the PM leads were successfully implanted into the right atrium and right ventricular apex (Figure 2). Review of the previous echocardiographic images confirmed these findings, showing the PLSVC traversing under the left atrium (LA) (Figure 3(a)) and inserting into the dilated coronary sinus (Figure 3(b)). The patient had an uneventful recovery and was discharged to an acute rehabilitation center for continued physical therapy.

## 2. Review of the Literature

**Figure 4:**
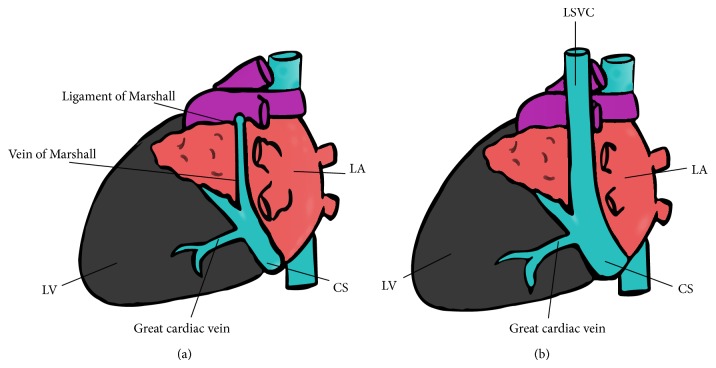
(a) Schematic diagram demonstrating normal venous return to the right atrium. The vein of Marshall regresses during embryologic development, forming the ligament of Marshall. (b) Schematic diagram demonstrating a persistent left superior vena cava which empties into a dilated coronary sinus. The PLSVC results from failure of the vein of Marshall to regress during embryologic development.

PLSVC is the most common thoracic venous anomaly and is a persistent congenital remnant of the vein of Marshall, which serves as a counterpart to the superior vena cava (SVC) in early embryologic development. This vein fails to regress as development continues and, as a result, venous blood returns to the RA via the connection of the PLSVC into the coronary sinus (Figure 4). Although it may be present in up to 0.5% of the general population, PLSVC, in the absence of other congenital cardiac anomalies, is almost never diagnosed because it tends to be hemodynamically insignificant, rarely leading to symptoms [1, 2]. However, it is encountered more commonly in patients undergoing placement of a PM and/or implantable cardiac defibrillators (ICDs); in one study of 300 patients with arrhythmias who underwent an electrophysiologic study prior to PM or ICD placement, approximately 4% had anomalies of venous drainage [1].

This higher prevalence of PLSVC detection during PM/ICD placement is likely due to the fact that patients with congenital anomalous venous return are at an increased risk of abnormalities associated with the cardiac conduction system, leading to arrhythmias that may require the placement of PM or defibrillators [3]. The embryologic pacemaker tissues of the heart are derived from two sites (right and left) near the progenitors of the superior vena cava (SVC). During normal development, the right sided site (located in the sinus venosus) usually forms the sinoatrial node while the left sided site (located in the posterior cardinal vein) migrates downward to an area near the coronary sinus. This latter (left sided) tissue in normal development loses its conduction ability as the vein degenerates, but it is retained if this tissue fails to regress and instead forms a PLSVC. Consequently, abnormal electrophysiologic function can arise from this site, manifesting as both tachyarrhythmias (supraventricular tachycardias, atrial fibrillation/flutter, or Wolff-Parkinson-White syndrome) and bradyarrhythmias (due to the development of atrioventricular conduction blocks) [1, 3]. It should also be noted that arrhythmias can arise from secondary causes as well, such as physiologic stresses placed on the conductive tissue as a result of the patients' abnormal anatomy that can lead to the enlargement of the right atrium or dilation of the coronary sinus [3].

In the majority of the cases in which PLSVC is discovered during or before the procedure, access is switched to the right subclavian vein, allowing for an easier route for lead navigation [4]. However, there are instances when the right sided approach is not a viable option, such as previous trauma to the patients' right side, time constraints in emergent situations, or even the congenital absence of a normal right sided SVC [5]. There are numerous case reports of successful implantation of various types of cardiac devices from the left subclavian approach in patients with PLSVC, including PM (single and dual chamber), ICD (single and dual chamber), and biventricular ICDs (which requires a third lead that traverses through the coronary sinus to the LV, providing right and left ventricular synchrony) [6–9].

With the recent advances in imaging, primary care providers can expect to see more patents in whom a PLSVC is incidentally diagnosed by computed tomography or magnetic resonance imaging that was performed for a variety of indications. Given the known association between anomalous venous return and the propensity for cardiac arrhythmias, this finding should be documented in the medical record and an inquiry made about possible cardiac symptoms, such as decreased exercise tolerance, progressive fatigue, chest discomfort, palpitations, or syncope. Annual clinical followup, including an electrocardiogram, should be continued and referral for formal cardiac evaluation should be prompted by any deviations from the patient's baseline [3]. It is important to be aware that tachycardias can occur despite PM placement and providers should consider this possibility if symptoms are consistent.

Proper understanding of the embryology and pathophysiology of PLSVC will reduce unnecessary and potentially harmful testing, premature subspecialty referral, and patient anxiety while providing optimal care for those few patients who truly need further evaluation and treatment.
